# Antiseizure Effects of *Peganum harmala* L. and *Lavandula angustifolia*

**DOI:** 10.1155/2023/4121998

**Published:** 2023-12-05

**Authors:** Zahra Rahimian, SeyedHassan Sadrian, Mina Shahisavandi, Hadi Aligholi, Mohammad M. Zarshenas, Alireza Abyar, Zahra Zeraatpisheh, Ali A. Asadi-Pooya

**Affiliations:** ^1^Epilepsy Research Center, Shiraz University of Medical Sciences, Shiraz, Iran; ^2^Students Research Committee, School of Medicine, Shiraz University of Medical Sciences, Shiraz, Iran; ^3^Department of Neuroscience, School of Advanced Medical Sciences and Technologies, Shiraz University of Medical Sciences, Shiraz, Iran; ^4^Department of Phytopharmaceuticals (Traditional Pharmacy), Faculty of Pharmacy and Pharmaceutical Sciences Research Center, Shiraz University of Medical Sciences, Shiraz, Iran; ^5^Medicinal Plants Processing Research Center, Shiraz University of Medical Sciences, Shiraz, Iran; ^6^Research Center for Psychiatry and Behavioral Sciences, Shiraz University of Medical Sciences, Shiraz, Iran; ^7^Jefferson Comprehensive Epilepsy Center, Department of Neurology, Thomas Jefferson University, Philadelphia, Pennsylvania, USA

## Abstract

*Peganum harmala* L. and *Lavandula angustifolia* are two traditional herbs with probable antiseizure effects. This study evaluated the effects of these two herbal extracts on pentylenetetrazol- (PTZ-) induced seizures in mice. We prepared hydroalcoholic extracts using *P. harmala* seeds and the aerial parts of *L. angustifolia* and then randomly divided 190 mice into 19 groups. Normal saline (10 mg/kg), diazepam (2 mg/kg), *P. harmala* (2.5, 5, 10, 15, 30, 45, and 60 mg/kg), and *L. angustifolia* (200, 400, 600, and 800 mg/kg) were intraperitoneally (IP) administrated 30 min before an IP administration of PTZ (90 mg/kg). Animals were observed for behavioral changes for one hour. In addition, the effects of flumazenil and naloxone on the antiseizure activity of *P. harmala* and *L. angustifolia* were assessed. *P. harmala* showed antiseizure activity at the dose of 10 mg/kg; it prolonged the seizure latency and decreased the seizure duration. The mortality protection rate was 90% for this herbal extract. *L. angustifolia* (600 mg/kg) prolonged the seizure latency and decreased both seizure duration and mortality. Neither flumazenil nor naloxone significantly reversed the antiseizure activities of *P. harmala* and *L. angustifolia.* In mice, the hydroalcoholic extracts of *P. harmala* and *L. angustifolia* showed antiseizure activity against PTZ-induced seizures. We could not delineate the exact antiseizure mechanisms of these extracts in the current study.

## 1. Introduction

About 70 million people have epilepsy worldwide, and most patients with epilepsy (PWE) live in low-income countries [1, 2]. The cornerstone of treating epilepsy is antiseizure medications (ASMs) [3]; however, about 30% of PWE may need additional therapeutic modalities like surgery due to suffering from uncontrolled, drug-resistant seizures [4, 5]. While surgery can be a beneficial treatment option for drug-resistant epilepsy, it is not always an option [5]. Hence, the need for novel medications or treatment methods for epilepsy is irrefutable [6].

Some herbal and traditional medications can potentially alleviate neuronal excitability [7]. *Peganum harmala* L., also known as the Espand, Harmel, African rue, or Mexican rue, is a medicinal plant with various pharmacological effects [8]. Different parts of *P. harmala,* such as its fruits, seeds, bark, and root, have been extensively used in Iran and other countries [9]. Due to its pharmacological activities, this herb has been used in traditional Persian medicine (TPM) for a long time [10, 11]. Most of the plant's beneficial effects are attributed to alkaloids such as harmaline and harmine [12]. However, this medicinal plant may give rise to some considerable adverse effects, especially following oral consumption [11]. Few studies have suggested an antiseizure feature for this plant extract, the details of which remain unclear [13].

Another famous and widely used herbal plant in Iran is the genus lavender, which has several beneficial effects on the central nervous system. It contains certain chemical compositions that improve conditions like depression and anxiety [14]. Great scientists like Avicenna and al-Razi also suggested that lavender possesses antiseizure properties besides its beneficial effects on pain and tremor [15–18].

*Lavandula angustifolia* Mill. (syn*: Lavandula officinalis*), also known as Ustukhuddoos, is one of the lavender species that traditional healers have extensively used for neurological diseases like epilepsy and migraines [19, 20]. In addition, some previous animal studies showed the antiseizure effects of this plant's hydroalcoholic extract at specific doses [20]. These properties have been attributed to linalool, a constituent with antidepressant and antiseizure effects [14].

The present study investigated the effects of hydroalcoholic extracts of *P. harmala* seeds and *L. angustifolia* aerial parts on seizures in a pentylenetetrazol- (PTZ-) induced seizure model in mice. We also investigated the involvement of opioid and benzodiazepine receptors in the antiseizure effects of these two herbs since some studies suggest the modulation of these receptors by *P. harmala* [9, 21] and *L. angustifolia* [22–24] extracts.

## 2. Materials and Methods

**Figure 1:**
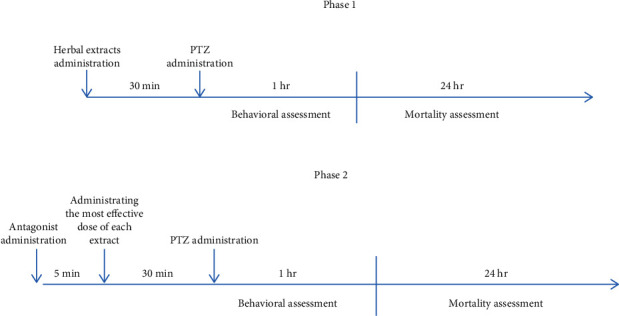
Description of each phase of study.

This study was conducted in two phases. First, antiseizure activities of *Peganum harmala L* and *Lavandula angustifolia* extracts were assessed separately through behavioral assessment. In the second or mechanistic phase, we evaluated the effect of flumazenil and naloxone as selective benzodiazepine and opiate receptor antagonists on the antiseizure activity of the most effective doses of each herbal extract (Table 1 and Figure 1).

### 2.1. Animals

One hundred and ninety Syrian male white mice (20-30 gr each) were obtained from the Comparative and Experimental Medicine Center of Shiraz University of Medical Sciences, Shiraz, Iran. We randomly divided the mice into 19 groups (*N* = 10/group). Every five animals were housed in one cage in a room at 22 ± 2°C with 45-55% humidity. They were housed under a 12 hr light/12 hr dark cycle with lights on at 6:00 in the morning. Water and ordinary food storage (Behparvar Co., Iran) were available ad libitum. The time of habituation of mice was one week. The animals were transferred to separate cages half an hour before the start of experimentation. All experiments were done between 8 a.m. and 11 a.m. Each animal was tested once. All experiments were conducted in compliance with the institutional guidelines for animal care in animal studies. The Ethics Committee of Shiraz University of Medical Sciences approved the study protocol (IR.SUMS.MED.REC.1400.071).

### 2.2. Plants and Extracts

*Peganum harmala* L. (voucher No. PM1397) seeds and *L. angustifolia* (voucher no. PM1302) aerial parts were collected from the medicinal plant garden of Shiraz School of Pharmacy. Samples of the herbs were deposited at the Herbarium of Shiraz School of Pharmacy with a specified authentication number. To extract with ethanol (70%), 100 gr of pulverized *P. harmala* seeds were subjected to an ultrasonic bath thrice, 20 min each time. The yielded extracts were subsequently concentrated, dried, and kept at 4°C. The same extraction method was repeated for the aerial parts of *L. angustifolia*.

### 2.3. Chemicals

Drugs used were as follows: PTZ (Sigma), flumazenil (Hameln, Germany), diazepam (EXIR, Iran), and naloxone (Tolid Daru Co., Iran). Diazepam was dissolved in 30% dimethyl sulphoxide (DMSO) in 0.9% NaCl with a drop (50 microliter) of Tween 80. PTZ and naloxone were dissolved in normal saline. All compounds were prepared each time freshly and administered intraperitoneally (IP).

### 2.4. Antiseizure Activity

In the first phase of the study, 13 mice groups were randomly selected. The mice received *P. harmala* (2.5, 5, 10, 15, 30, 45, or 60 mg/kg, IP), *L. angustifolia* (200, 400, 600, or 800 mg/kg IP), diazepam (2 mg/kg), or normal saline (10 ml/kg) 30 minutes prior to the IP administration of PTZ (90 mg/kg) [25]. Then, behavioral changes of the mice were assessed for 1 hour. Extract doses were based on pilot experiments and previous studies [13, 20, 26].

### 2.5. Behavioral Assessments

Following PTZ injection, each mouse was placed in a Plexiglas cage and was observed for one hour to assess seizure behaviors. The categorization of PTZ-induced seizure behaviors was explained in a previous study [27]: no seizure behavior: score 0; restlessness: score 1; motionless staring: score 2; hind-limb tonic extension/Straub's tail: score 3; myoclonic jerk: score 4; tonic-clonic seizure: score 5. The time (seconds) between PTZ injection and the onset of the hind-limb tonic extension was considered as the seizure latency. The commencement of a tonic-clonic seizure was considered as the tonic-clonic onset. The duration of the first attack of the tonic-clonic phase was considered as the seizure duration. Furthermore, the time of death of each mouse was recorded and reported as death time. Finally, the mortality protection rate was evaluated 24 hours after PTZ injection. The observation process was completed by an expert observer who was blind to the identity of all study groups.

### 2.6. Effects of Flumazenil on the Antiseizure Activity of Herbal Extracts

To investigate the probable involvement of benzodiazepine (BDZ) receptors in the antiseizure activity of *P. harmala* and *L. angustifolia* [28], we performed further experiments in accordance with a previous study [29]. The best doses of the extracts were selected according to the antiseizure activity phase of the study (see Section 2.4). We examined the effects of flumazenil, as a selective benzodiazepine receptor antagonist, on the antiseizure activity of *P. harmala* and *L. angustifolia*. The administration time of the antagonist before the antiseizure treatment was five minutes. Animals were administered normal saline (10 ml/kg) (see Section 2.4), diazepam (2 mg/kg) (see Section 2.4), *P. harmala* (10 mg/kg) (see Section 2.4), *L. angustifolia* (600 mg/kg) (see Section 2.4), diazepam + flumazenil, *P. harmala*+flumazenil, or *L. angustifolia*+flumazenil IP, 30 minutes before the IP administration of 90 mg/kg PTZ. Behavioral assessments were done for 1 hour.

### 2.7. Effects of Naloxone on the Antiseizure Activity of the Herbal Extracts

The same strategy as in Section 2.6 (as for flumazenil) was followed, and 5 mg/kg naloxone [29] (instead of flumazenil) was administered alone or with the extracts of *P. harmala* (10 mg/kg) or *L. angustifolia* (600 mg/kg) 30 minutes before the IP administration of PTZ (90 mg/kg).

### 2.8. Statistical Analysis

We used IBM SPSS version 26 for the data analyses. Data were presented as mean ± standard deviation (SD). Data related to each herbal extract was analyzed distinctly. The normality of data distribution was tested through the Kolmogorov-Smirnov test. Statistical analysis, for evaluating both phases of the study, was carried out using one-way analysis of variance flowed by Tukey's post hoc test for parametric data. Also, the Kruskal-Wallis test with Dunn's post hoc test was applied for the analysis of nonparametric data. The Mann–Whitney *U* test was applied where comparisons of two groups were needed (to compare death time in phase two of the study). The significance level was considered *P* < 0.05.

## 3. Results

### 3.1. Antiseizure Activity of *Peganum harmala* L. Extract

As illustrated in Table 2, the administration of investigated doses of *P. harmala* extract significantly affected seizure latency (*H* = 43.393, *P* < 0.001), tonic-clonic onset (*H* = 27.638, *P* < 0.001), seizure duration (*H* = 42.330, *P* < 0.001), and death time (*H* = 21.730, *P* < 0.05). Also, the post hoc tests analysis indicated that the injection of *P. harmala* extract significantly prolonged the seizure latency at the doses of 2.5, 5, and 10 mg/kg (*P* < 0.05). In addition, seizure duration was significantly reduced at these three doses compared with the saline group (*P* < 0.001). Furthermore, 10 mg/kg of *P. harmala* hydroalcoholic extract had the highest mortality protection rate.

### 3.2. Effect of Flumazenil on the Antiseizure Activity of *Peganum harmala* L. Extract

**Figure 2:**
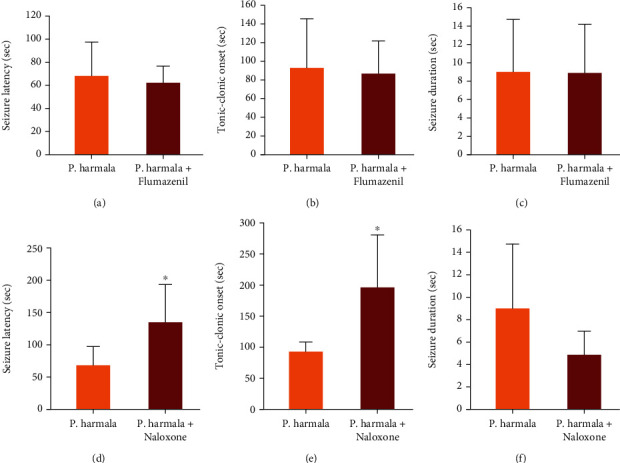
Effect of flumazenil (a–c) and naloxone (d–f) on the antiseizure activity of *Peganum harmala* L. in pentylenetetrazol- (PTZ-) induced seizures in mice. ^∗^*P* < 0.05 refers to post hoc analyses that compare these two groups ((a–c) Tukey's post hoc test; (d–f) Dunn's test for pairwise comparison).

Administration of flumazenil (2 mg/kg) five minutes before the injection of 10 mg/kg *P. harmala* extract did not reverse the antiseizure activity of the *P. harmala* extract in terms of the seizure latency, the onset of the tonic-clonic phase, the seizure duration, or the mortality protection rate (Table 3 and Figure 2). Furthermore, there was a significant difference in the seizure latency and seizure duration among mice that received *P. harmala (*10 mg/kg) pretreated with flumazenil and the group which received normal saline (Table 3). Also, flumazenil reversed the anticonvulsant activity of diazepam (Table 3).

### 3.3. Effect of Naloxone on the Antiseizure Activity of *Peganum harmala* L. Extract

Pretreatment of mice with 5 mg/kg naloxone five minutes before the injection of 10 mg/kg *P. harmala* extract caused significant prolongation in seizure latency and tonic-clonic phase onset (*P* < 0.05). However, seizure duration and mortality protection were not significantly affected by naloxone (Table 4 and Figure 2). Other findings were that the differences in the seizure latency, tonic-clonic onset, and seizure duration among mice that received *P. harmala (*10 mg/kg) pretreated with naloxone (5 mg/kg) and the saline group were statistically significant (Table 4).

### 3.4. Antiseizure Activity of *Lavandula angustifolia* Extract

As shown in Table 5, the administration of studied doses of *L.angustifolia* extract significantly affected seizure latency (*H* = 18.918, *P* < 0.05), tonic-clonic onset (*F* = 5.445, *P* = *P* < 0.05), seizure duration (*H* = 20.659, *P* < 0.001), and death time (*H* = 17.503, *P* = *P* < 0.05). Also, the post hoc test analysis indicated that the injection of 200, 400, or 600 mg/kg of *L.angustifolia* extract significantly prolonged the seizure latency and tonic-clonic onset. In addition, 200 mg/kg and 600 mg/kg of the extract led to a significant reduction in seizure duration compared to the saline group (*P* < 0.05). This extract had a 90% mortality protection rate at the dose of 600 mg/kg.

### 3.5. Effects of Flumazenil on the Antiseizure Activity of *Lavandula angustifolia* Extract

**Figure 3:**
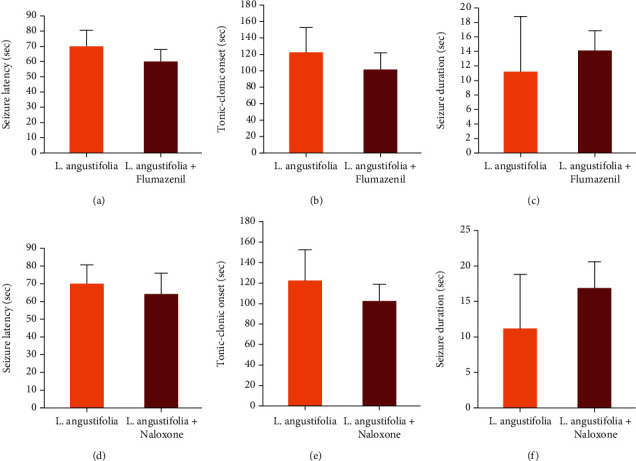
Effect of flumazenil (a–c) and naloxone (d–f) on the antiseizure activity of *Lavandula angustifolia* in pentylenetetrazol- (PTZ-) induced seizures in mice. No significant difference was observed among these groups in post hoc test analyses ((a, c) Dunn's test for pairwise comparison and (b, d–f) Tukey's post hoc test).

Administration of 2 mg/kg flumazenil five minutes before the *L. angustifolia* extract (600 mg/kg) injection did not reverse the effect of *L. angustifolia* extract on seizure latency, tonic-clonic phase onset, seizure duration, or mortality protection (Table 6 and Figure 3). Also, as indicated in Table 6, there were significant differences in the seizure latency, tonic-clonic onset, and seizure duration in mice which received *L. angustifolia* (600 mg/kg) pretreated with flumazenil and the group that received normal saline (Table 6). Furthermore, flumazenil could reverse the anticonvulsant activity of diazepam (Table 6).

### 3.6. Effects of Naloxone on the Antiseizure Activity of *Lavandula angustifolia* Extract

Pretreatment of mice with 5 mg/kg of naloxone five minutes prior to the injection of 600 mg/kg *L. angustifolia* extract did not reverse the effect of *L. angustifolia* extract on the seizure latency, onset of tonic-clonic phase, seizure duration, or mortality protection (Table 7 and Figure 3). Furthermore, significant differences were detected in seizure latency, tonic-clonic onset, and seizure duration among mice that received *L. angustifolia* (600 mg/kg) pretreated with naloxone and the saline group (Table 7).

## 4. Discussion

In recent years, investigations about the medicinal properties of plants have been undertaken by scientists worldwide due to their pharmacological potency and cost-efficiency [30]. The current study investigated the antiseizure effects of *P. harmala* and *L. angustifolia* as two herbal medicines with various health benefits. Our study revealed that *P. harmala* could prolong the appearance of seizure behaviors. In addition, it could suppress the duration of clonic seizures. The dose of 10 mg/kg of this extract also protected most mice against death. It seems that low doses of *P. harmala* extract have antiseizure activity, while higher doses do not. This study also indicated that 600 mg/kg of *L. angustifolia* extract exhibited the most prolonged seizure latency, the lowest seizure duration, and the most mortality protection compared with other doses. *L. angustifolia* prolonged seizure latency and caused a delay in the clonic convulsion at most doses.

Our study is in agreement with the survey designed by Hashemi et al. about the antiepileptic features of *P. harmala* seed extract. The researchers examined the effects of 15, 30, and 45 mg/kg of the methanol extract of *P. harmala* seeds on seizure latency, seizure duration, and death rate in 120 Albion Swiss male mice. The study indicated that oral administration of the extract (45 mg/kg) could increase the seizure latency while causing a 58% reduction in the mortality rate. Duration of seizure was another variable that was significantly affected by 45 mg/kg of methanol extract of *P. harmala* seeds, while the other doses had no significant effect on these variables [13]. However, our study indicated that lower doses of the extract offered antiseizure effects, which may be due to the IP administration route instead of the oral route used in the Hashemi et al. study.

Various mechanisms have been postulated to explain both epileptogenic and antiseizure effects of *P. harmala* [13, 31]. Phytochemical qualitative analysis of this plant seed extract revealed the presence of alkaloids, flavonoids, and anthraquinones [32]. Four new flavonoids including acacetin 7-O-rhamnoside, 7-O-(6^″^-O-glucosyl-2^″^-O-(3^″′^-acetylrhamnosyl) glucoside, 7-O-(2^″′^-O-rhamnosyl-2^″^-O-glucosylglucoside), and glycoflavone 2^″′^-O-rhamnosyl-2^″^-O-glucosylcytisoside have been detected in the aerial parts of the plant [33]. The total alkaloid content of this plant varies from 2 to 5%, with its seeds offering the highest amount [10, 34]. The main beta-carboline alkaloids in the seeds are harmaline and harmine [10, 34]. Harmaline seems to play a fundamental role in many pharmacological activities of *P. harmala* [9]. The study of Alenajaf et al. about the effects of harmaline on seizures induced by amygdala kindling in rats illustrated that IP injection of 15 mg/kg of harmaline had a significant proseizure effect in fully kindled rats. That study suggested that harmaline can augment neuronal activity through stimulation of NMDA receptors and inverse agonist activity at GABA receptors [31]. Also, some records claimed that simultaneous IP injection of harmaline and PTZ could lower the seizure threshold in a dose-dependent manner [26]. This mechanism may be the cause of the nonprotective effects of higher doses of *P. harmala* seen in our study.

The present study indicated that naloxone increased the prolongation of seizure onset and decreased the onset of the tonic-clonic phase induced by *P. harmala*. Palizvan and Ghazvavi-Rad demonstrated that verapamil and naloxone had synergic effects on seizure inhibition in the PTZ kindling model of rats. This study suggested that verapamil, as an L-type calcium channel blocker, and naloxone, as an opiate receptor antagonist, had synergic effects in controlling seizures induced by PTZ [35]. Harmaline also provides a muscle relaxant effect by inhibiting L- and N-type calcium channels [36–38]. This suggests that one of the mechanisms behind the antiseizure properties of *P. harmala* extract may be L-type calcium channel blocking, explaining its synergic antiseizure interaction with naloxone.

In this study, flumazenil, as an antagonist of benzodiazepine receptors, did not antagonize the antiseizure activity of *P. harmala* extract in the PTZ model of mice. Hence, the antiseizure effect of *P. harmala* does not seem to be associated with benzodiazepine receptor activation.

Some previous studies also reported the antiseizure effects of *L. angustifolia* extract. Rahmati et al. conducted a study in which they examined the effect of *L. angustifolia* hydroalcoholic extract (200, 400, or 800 mg/kg IP) on the progression of seizure stages, seizure latency, and duration of phases two and five of seizures in mice. In that study, kindling was done by injecting 30 mg/kg of PTZ. The results showed that the dose of 200 mg/kg of *L. angustifolia* had a significant influence on lowering the score and duration of the seizure [39]. Another study on nicotine-induced seizures in mice conducted by Arzi et al. indicated that 600 mg/kg of *L. angustifolia* hydroalcoholic extract was the most effective dose, significantly influencing the intensity, onset, and duration of the seizure. Accordingly, our study also found that the dose of 600 mg/kg of *L. angustifolia* extract was the most effective dose in controlling seizure onset and duration. Also, it protected most animals against mortality [20].

The presence of tannins, coumarins, flavonoids, volatile oils, and fatty acids has been confirmed through phytochemical screening of the aerial parts of *L. angustifolia* [40]. Our study indicated that injecting 2 mg/kg of flumazenil five minutes before administering 600 mg/kg of *L. angustifolia* hydroalcoholic extract could not significantly antagonize the extract's effect on seizure protection. This is consistent with the study of Lopez et al., indicating that *L. angustifolia* has no affinity for GABAA-benzodiazepine receptors [14]. We also found that the antiseizure activity of the lavender extract was not significantly affected by naloxone as an opioid receptor antagonist, suggesting that the opioid system is not involved in the antiseizure effects of the extract.

### 4.1. Limitations

In the current study, two plant extracts showed efficacy to control seizures induced by PTZ (90 mg/kg) as a stringent seizure assay. However, two limitations should be considered. First, this study could not identify the main extract compounds responsible for ameliorating PTZ-induced seizures. Second, we only examined the involvement of benzodiazepine and opioid receptors, while other mechanisms like the involvement of glutamate receptors were not evaluated.

## 5. Conclusion

The current study provides evidence in favor of the antiseizure activity of *P. harmala* and *L. angustifolia* hydroalcoholic extracts in the PTZ model of seizures. The most effective doses were 10 mg/kg and 600 mg/kg of *P. harmala* and *L. angustifolia,* respectively. Moreover, these two plant extracts did not act via activation of benzodiazepine or opioid receptors. Further studies should be designed to detect the mechanisms behind the antiseizure properties of these plants.

## Figures and Tables

**Table 1:** An overall overview of the study design.

*Peganum harmala* L. extract	**Phase 1** (i) Assessing antiseizure activity of seven doses of *P. harmala* (ii) Variables were compared between *P. harmala* extract in different doses and normal saline group
	**Phase 2** (i) Mechanistic phase (ii) Antiseizure activity of the most effective dose of *Peganum harmala* L (10 mg/kg) was compared with *Peganum harmala* L+ each antagonist

*Lavandula angustifolia* extract	**Phase 1** (i) Assessing antiseizure activity in four doses of *L. angustifolia* (ii) Variables were compared between *L. angustifolia* extract in different doses and normal saline group
	**Phase 2** (i) Mechanistic phase (ii) Antiseizure activity of the most effective dose of *L. angustifolia* (600 mg/kg) was compared with *L. angustifolia*+each antagonist

**Table 2:** Effects of *Peganum harmala* L. on seizures induced by pentylenetetrazol (PTZ) in mice^a^.

Treatment	Dose	Seizure latency (sec)	Tonic-clonic onset (sec)	Seizure duration (sec)	Death time (sec)	Mortality protection rate (%)
Normal saline	10 ml/kg	32.30 ± 17.04	51.60 ± 23.83	38.00 ± 15.49	290.50 ± 135.98	0
Diazepam	2 mg/kg	—	—	—	—	100
*Peganum harmala*	2.5 mg/kg	52.90 ± 10.21^∗^	79.56 ± 34.78	11.44 ± 7.92^∗∗∗^	618.75 ± 245.98	60
	5 mg/kg	53.50 ± 8.00^∗^	82.60 ± 31.97	8.70 ± 5.55^∗∗∗^	802.50 ± 362.80^∗^	60
	10 mg/kg	68.10 ± 29.35^∗∗∗^	92.86 ± 52.53^∗^	9.00 ± 5.74^∗∗∗^	—	90
	15 mg/kg	45.40 ± 51.71	88.10 ± 84.94	60.00 ± 32.19	268.00 ± 144.87	20
	30 mg/kg	32.00 ± 6.79	42.40 ± 8.65	29.80 ± 17.63	277.77 ± 233.79	10
	45 mg/kg	34.50 ± 4.30	45.90 ± 6.36	28.20 ± 32.45	184.00 ± 117.80	10
	60 mg/kg	33.20 ± 7.88	60.20 ± 15.72	44.10 ± 70.34	257.20 ± 156.02	0
*P* value	—	0.000^#^	0.000^#^	0.000^#^	0.003^#^	—
Test statistic	—	43.393^#^	27.638^#^	42.330^#^	21.730^#^	—

^a^Normal saline, diazepam, and *Peganum harmala* L. were administered 30 minutes prior to PTZ injection (90 mg/kg). ^#^*P* values and test statistics (*H*) refer to the Kruskal-Wallis test and assess differences in each variable among studied groups. ^∗^*P* < 0.05 and ^∗∗∗^*P* < 0.001 compared with the normal saline group; refer to the post hoc tests (Dunn's test).

**Table 3:** Effect of flumazenil on the antiseizure activity of *Peganum harmala* L. and diazepam in pentylenetetrazol- (PTZ-) induced seizures in mice^a^.

Treatment	Dose	Seizure latency (sec)	Tonic-clonic onset (sec)	Seizure duration (sec)	Death time (sec)	Mortality protection rate (%)
Normal saline	10 ml/kg	32.30 ± 5.38	51.60 ± 7.53	38 ± 4.90	290.50 ± 135.98	0
Diazepam	2 mg/kg	—	—	—	—	100
Diazepam+flumazenil	2 mg/kg, 2 mg/kg	59.50 ± 11.56	75.60 ± 12.72	21.5 ± 5.58	—	90
*P. harmala*	10 mg/kg	68.10 ± 29.35^∗∗∗^	92.86 ± 52.53^∗^	9.00 ± 5.74^∗∗∗^	—	90
*Peganum harmala*+flumazenil	10 mg/kg, 2 mg/kg	62.20 ± 14.48^∗^	86.70 ± 34.96	8.90 ± 5.30^∗∗∗^	—	90
*P* value	—	0.001^+^	0.048^+^	0.000^+^	—	—
Test statistic	—	6.756^+^	2.924^+^	20.302^+^	—	—

^+^ *P* values and test statistics (*F*) refer to one-way ANOVA test. ^∗^*P* < 0.05 and ^∗∗∗^*P* < 0.001 compared with the normal saline group; refer to Tukey's post hoc test. ^a^The results of post hoc analyses that compare the effects of *Peganum harmala* and *Peganum harmala*+flumazenil are indicated in Figure 2.

**Table 4:** Effect of naloxone on the antiseizure activity of *Peganum harmala* L. in pentylenetetrazol- (PTZ-) induced seizures in mice^a^.

Treatment	Dose	Latency to seizure onset (sec)	Tonic-clonic onset (sec)	Seizure duration (sec)	Death time (sec)	Mortality protection rate (%)
Normal saline	10 ml/kg	32.30 ± 5.38	51.60 ± 7.53	38 ± 4.90	290.50 ± 135.98	0
Naloxone	5 mg/kg	39.50 ± 10.59	76.60 ± 16.40	28.00 ± 6.53	219.00 ± 95.47	20
*P. harmala*	10 mg/kg	68.10 ± 29.35^∗∗∗^	92.86 ± 52.53^∗^	9.00 ± 5.74^∗∗∗^	—	90
*P. harmala+*naloxone	10 mg/kg, 5 mg/kg	135.10 ± 58.59^∗∗∗^	196.12 ± 84.53^∗∗∗^	4.87 ± 2.10^∗∗∗^	—	90
*P* value	—	0.000^#^	0.000^#^	0.000^#^	0.102^##^	—
Test statistic	—	26.572^#^	20.623^#^	25.344^#^	25.000^##^	—

^#^ *P* values and test statistics (H) refer to the Kruskal-Wallis test. ^##^*P* values and test statistics (U) refer to the Mann–Whitney *U* test. ^∗^*P* < 0.05 and ^∗∗∗^*P* < 0.001 compared with the normal saline group; refer to the post hoc tests (Dunn's test). ^a^The results of post hoc analyses that compare the effects of *Peganum harmala* and *Peganum harmala*+naloxone are indicated in Figure 2.

**Table 5:** Effect of *Lavandula angustifolia* on seizures induced by pentylenetetrazol (PTZ) in mice.

Treatment	Dose (mg/kg)	Seizure latency (sec)	Tonic-clonic onset (sec)	Seizure duration (sec)	Death time (sec)	Mortality protection (%)
Normal saline	10 ml/kg	32.30 ± 5.38	51.60 ± 7.53	38 ± 4.90	290.50 ± 135.98	0
Diazepam	2 mg/kg	—	—	—	—	100
*L. angustifolia*	200 mg/kg	78.80 ± 47.34^∗∗∗^	120.00 ± 59.88^∗^	19.20 ± 8.20^∗^	340.66 ± 169.24	40
	400 mg/kg	68.30 ± 22.08^∗∗∗^	112.30 ± 44.45^∗^	31.40 ± 16.66	683.44 ± 323.93^∗^	10
	600 mg/kg	70.00 ± 10.59^∗∗∗^	122.5 ± 30.14^∗∗∗^	11.2 ± 7.62^∗∗∗^	—	90
	800 mg/kg	55.40 ± 15.24	86.10 ± 34.41	29.90 ± 10.54	1106.50 ± 396.28^∗^	60
*P* value	—	0.001^#^	0.001^+^	0.000^#^	0.002^#^	—
Test statistic	—	18.918^#^	5.445^+^	20.659^#^	17.503^#^	—

^a^Normal saline, diazepam, and *L. angustifolia* were administered 30 minutes prior to PTZ injection (90 mg/kg). ^#^*P* values and test statistics (*H*) refer to the Kruskal-Wallis test. ^+^*P* values and test statistics (*F*) refer to one-way ANOVA test. ^∗^*P* < 0.05 and ^∗∗∗^*P* < 0.001 compared with the normal saline group; refer to the post hoc tests (Dunn's test for the Kruskal-Wallis test and Tukey's test for one-way ANOVA).

**Table 6:** Effect of flumazenil on the antiseizure activity of *Lavandula angustifolia* and diazepam in pentylenetetrazol- (PTZ-) induced seizures in mice^a^.

Treatment	Dose (mg/kg)	Latency to seizure onset (sec)	Tonic-clonic onset (sec)	Seizure duration (sec)	Death time (sec)	Mortality protection rate (%)
Normal saline	10 ml/kg	32.30 ± 5.38	51.60 ± 7.53	38 ± 4.90	290.50 ± 135.98	0
Diazepam	2 mg/kg	—	—	—	—	100
Diazepam+flumazenil	2 mg/kg, 2 mg/kg	59.50 ± 11.56	75.60 ± 12.72	21.5 ± 5.58	—	90
*L. angustifolia*	600 mg/kg	70.00 ± 10.59^∗∗∗^	122.5 ± 30.14^∗∗∗^	11.2 ± 7.62^∗∗∗^	—	90
*L. angustifolia*+flumazenil	600 mg/kg, 2 mg/kg	60.00 ± 7.87^∗^	101.6 ± 20.22^∗∗∗^	14.11 ± 2.75^∗^	330.00 ± 51.96	50
*P* value	—	0.001^#^	0.000^+^	0.000^#^	0.269^##^	—
Test statistic	—	17.534^#^	18.580^+^	23.930^#^	16.000^##^	—

^#^ *P* values and test statistics (H) refer to the Kruskal-Wallis test. ^+^*P* values and test statistics (*F*) refer to one-way ANOVA test. ^##^*P* values and test statistics (*U*) refer to the Mann–Whitney *U* test. ^∗^*P* < 0.05 and ^∗∗∗^*P* < 0.001 compared with the normal saline group; refer to the post hoc tests (Dunn's test for the Kruskal-Wallis test and Tukey's test for one-way ANOVA). ^a^The results of post hoc analyses that compare the effects of *L. angustifolia* and *L. angustifolia*+flumazenil are indicated in Figure 3.

**Table 7:** Effect of naloxone on the antiseizure activity of *Lavandula angustifolia* in pentylenetetrazol- (PTZ-) induced seizures in mice^a^.

Treatment	Dose (mg/kg)	Seizure latency (sec)	Tonic-clonic onset (sec)	Seizure duration (sec)	Death time (sec)	Mortality protection rate (%)
Normal saline	10 ml/kg	32.30 ± 5.38	51.60 ± 7.53	38 ± 4.90	290.50 ± 135.98	0
Naloxone	5 mg/kg	39.50 ± 10.59	76.60 ± 16.40	28.00 ± 6.53	219.00 ± 95.47	20
*L. angustifolia*	600 mg/kg	70.00 ± 10.59^∗∗∗^	122.5 ± 30.14^∗∗∗^	11.2 ± 7.62^∗∗∗^	—	90
*L. angustifolia*+naloxone	600 mg/kg, 5 mg/kg	64.20 ± 11.7^∗∗∗^	102.50 ± 16.41^∗∗∗^	17.75 ± 2.91^∗∗∗^	238.00 ± 102.99	60
*P* value	—	0.000^+^	0.000^+^	0.000^+^	0.401^+^	—
Test statistic	—	20.752^+^	18.889^+^	15.433^+^	0.954^+^	—

^+^ *P* values and test statistics (*F*) refer to one-way ANOVA test. ^∗∗∗^*P* < 0.001 compared with the normal saline group; refer to the post hoc tests (Tukey's post hoc test). ^a^The results of post hoc analyses that compare the effects of *L. angustifolia* and *L. angustifolia*+naloxone are indicated in Figure 3.

## Data Availability

Data of the study can be requested from the authors. Please write to the corresponding author if you are interested in such data.
